# Randomized in situ evaluation of surface polishing protocols on the caries-protective effect of resin Infiltrant

**DOI:** 10.1038/s41598-022-25091-8

**Published:** 2022-11-30

**Authors:** Marcella Esteves-Oliveira, Vanara F. Passos, Tereza M. A. Z. C. Russi, Argus R. R. Fernandes, Caroline N. N. Terto, Juliano S. Mendonça, Guglielmo Campus, Richard J. Wierichs, Hendrik Meyer-Lueckel, Juliana P. M. Lima

**Affiliations:** 1 Department of Restorative, Preventive & Pediatric Dentistry, University of Bern, Bern, Switzerland; 2 Department of Restorative Dentistry, Federal University of Ceara, Fortaleza, Ceara Brazil; 3 Christus University Center, Fortaleza, Ceará Brazil; 4 Department of Restorative Dentistry and Endodontology, Justus Liebig University Gießen, Gießen, Germany

**Keywords:** Medical research, Dental diseases

## Abstract

The aim of this placebo-controlled randomized in situ study was to evaluate the effect of different surface polishing protocols on enamel roughness, bacterial adhesion and caries-protective effect of a resin infiltrant. Seventy-five bovine enamel samples having artificial caries lesions were treated with a resinous infiltrant and afterwards randomly dividided into five polishing protocols: aluminum oxide flexible disks (Al_2_O_3_-Disks), silicon carbide tips (SIC-Tips), silicon carbide brush (SIC-Brush), silicon carbide polyester strips (SIC-Strips) or no polishing [negative control (NC)]. Average surface roughness (Ra) was assessed by profilometry. Samples were mounted in palatal appliances under a mesh for biofilm accumulation. Fifteen volunteers wore the intraoral appliances (14-days) and cariogenic challenge was triggered by sucrose solutions**.** Biofilm formed was collected for microbiological analysis of caries-related bacteria (*Streptococcus mutans, Lactobacillus acidophilus*) and demineralization was assessed by cross-sectional microhardness. Mean Knoop hardness numbers (Kg/mm^2^) were plotted over lesion depth (µm) and area under the lesion curve was subtracted from sound enamel to determine demineralization (ΔS, Kg/mm^2^xµm). Data were analyzed by ANOVA and *post-hoc* comparisons (α = 0.05). NC resulted in significantly higher Ra means than Al_2_O_3_-Disks and SIC-Strips. Bacterial counts were not significantly different between the groups (p > 0.05). Regards ΔS means, however none of the groups were significantly different to NC (6983.3 kg/mm^2^xµm /CI 4246.1–9720.5, p > 0.05). Conclusions: Polishing protocols (Al_2_O_3_-Disks, SIC-Strips) significantly decreseased roughness of infiltrated-enamel, however none of the polishing protocols could signicantly decrease bacterial counts nor resulted in significant less demineralization.

## Introduction

Currently a more biological approach to caries management is at least scientifically widely accepted. The understanding of caries as a multifactorial disease driven by a dysbiosis in the biofilm, the so called ecological plaque hypothesis, supports more holistic approaches for disease management including effective mechanical plaque control, diet modification and modulation of the remineralization^1^. Regarding the management of approximal non-cavitated caries lesions, the most actual consensus statements, systematic reviews and meta-analyses recommend both non- and micro-invasive treatment options^2–4^. The improvement of biofilm control via oral hygiene instructions and professional fluoride application (i.e., varnish) is a main component of the non-invasive approach. For patients at low caries risk, these measures should be enough to control the disease, yet, for patients at high caries risk, a higher probability and velocity of lesion progression might be expected, and in these cases the micro-invasive treatment options, like caries sealing and caries infiltration, have been recommended^2^.

However up to now, not much discussion has been provided on the susceptibility of infiltrated lesions to secondary caries or lesion progression due to increased plaque stagnation. Concurrently the control of biofilm formation or the predisposition of material surfaces, such as restorations, sealants and infiltrants to accumulate biofilm is known to play a significant role in caries control. Specially as surface irregularities in materials not only predispose for higher biofilm accumulation^5,6^, but also act like niches where biofilm may grow undisturbed from mechanical actions of occlusal function, friction from cheeks, tongue, and oral hygiene measures^7^.

A recent systematic review also confirms that considering composite resins polishing techniques may decrease bacterial adhesion through decrease of surface roughness^8^. Besides surface roughness, several surface properties can influence bacterial adhesion to restorative or sealing/infiltrant materials surfaces, *i.e.* surface free energy (surface wettability), charge, polarity, and morphology^6^. However, surface roughness seems to be the most important and most studied one, especially as regards the accumulation and composition of the biofilm^7,9^. Moreover, some years ago, a threshold value for average surface roughness (Ra) of 0.2 µm has been proposed, indicating that dental material surfaces with roughness means below this value should have no impact on bacterial adhesion^10^. However, this concept has been contested recently, since the influence of roughness on bacterial adhesion seems to be more related to roughness range rather than a threshold^11^. In addition factors like type of material and study design may also influence bacterial adhesion, with studies including formation of salivary pellicle, in situ or in vivo, having a clear advantage of better simulating the clinical conditions, where early colonizers first attach to salivary pellicle and the latter may also modify the surface of properties of the materials^6,12^.

Incipient/white-spot carious lesions are known to show higher roughness than sound enamel, due to increased episodes of enamel dissolution^13^ and resin infiltration of these lesions indeed decreases their surface roughness. Nevertheless, infiltrated caries lesions still have significantly higher roughness than non-treated sound enamel^14^. After the hydrochloric acid etching, opening of the lesion micropores and the lesion body for the infiltration of low-viscosity monomers, an increased lesion surface roughness is present^15^. The resin infiltration afterwards has been shown to effectively fill up this micro-porosities^16^, however without recovering of the initial roughness values^15,17^. As increased surface roughness among other factors play an important role in dental plaque accumulation and biofilm formation^5,6,12^, which in turn can favour the occurrence of caries adjacent to restorations and sealants (CARS)^3^, this factor might be of clinical relevance for the long-term treatment survival rate. This leads to the question of, whether surface polishing procedures, a common clinical step in restorative treatment, may be also indispensable for the long-term success of caries infiltration and prevention of caries around the infiltrated lesions as well as a more holistic caries control.

Currently, only one study have investigated the influence of polishing on the recovery of enamel surface roughness after resin infiltration of incipient carious lesions^15^. The polishing of infiltrated lesions, solely with one type of polishing strip showed no significant benefit regarding surface roughness and as compared to the non-polished infiltrated lesion^15^; even if the effect of the increased lesion surface roughness on bacterial adhesion was not tested. In fact, bacterial adhesion has also been tested only in one study including resin infiltration of incipient carious lesion, but without any polishing procedure after resin infiltration^18^.

In summary there is clear evidence that resin infiltration prevents caries progression clinically, but also that it increases lesion surface roughness, which (at least in theory) favours higher plaque accumulation. Until now no clinical recommendation has been made in the current clinical guidelines^19^, meta-analyses^2^ and consensus reports^3^ as regards how infiltrated approximal and buccal non-cavitated caries lesions should be polished in order to decrease surface roughness, plaque accumulation and the consequent increased risk of developing both gingivitis and secondary caries. Therefore, it is still unclear if certain polishing protocols after carious lesion infiltration with low-monomers infiltrant, could further considerably decrease enamel surface roughness as compared to non-polished infiltrated lesions and additionally decrease bacterial adhesion. Thus, the aim of this placebo-controlled randomized in situ study was to evaluate the effect of different surface polishing protocols on enamel surface roughness, bacterial adhesion and caries-protective effect of an infiltrant. It was thus hypothesized that polishing of resin-infiltrated incipient carious lesions would cause for all protocols a decrease in average surface roughness, adhesion of caries-associated bacteria and less in situ demineralization than the non-polished negative control.

## Materials and methods

This study was conducted according to the principles of the declaration of Helsinki and all experimental protocols were approved by the local Research and Ethical Committee of the Christus University Center (Protocol # 2758611). All participants signed a written informed consent before the start of the study.

### Study design and subjects

**Figure 1:**
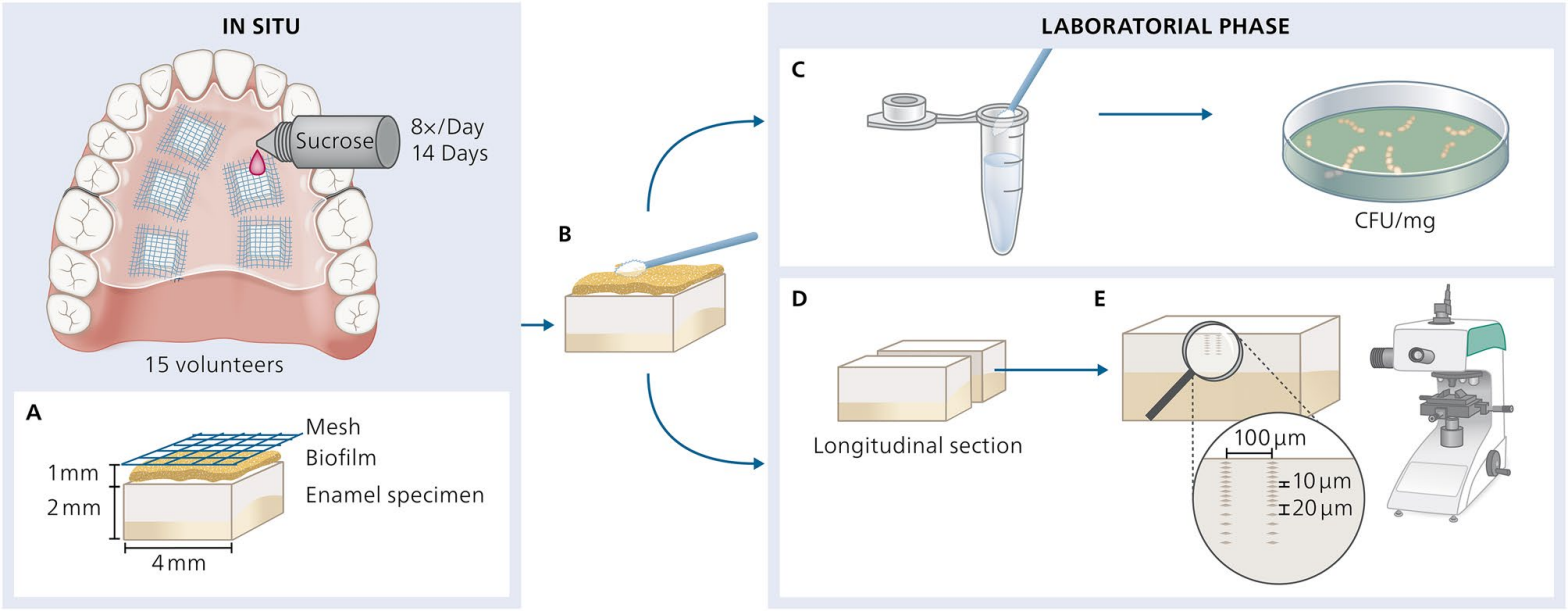
Study Design—Palatal appliance worn by the volunteers containing 5 bovine enamel specimens (4 × 4 × 2 mm^3^) with artificial caries lesions infiltrated with a resin infiltrant. Specimens were rinsed with sucrose 8 times/day for 14 days. (**A**) Mesh net placed 1 mm over the specimens for allowing plaque accumulation. (**B**) Specimens removed from the appliances after 14 days. (**C**) Biofilm collection from one specimen/appliance (n = 15/group) and determination of CFU/mg of total bacteria*, S. mutans and Lactobacillus acidophilius*. (**D**) Specimens sectioned longitudinally trough the center of the caries lesions (n = 1 specimen/group/appliance and n = 15 specimens/group). (**E**) Cross-sectional Knoop microhardness analysis. Two lines of indentations 100 µm apart from each other and up to a depth of 180 µm. Indentation interval was 10 µm up to 100 µm depth and 20 µm from 100 to 180 µm, so that 14 indentations were performed per line. (Drawing of Ms. Bernadette Rawyler).

This study was a single-center, placebo-controlled, double-blinded (volunteer and examiner) randomized in situ study composed of one single- factor (polishing types), at 5 levels (polishing protocols). Randomization and blinding were performed by the supervisor of the study using the random sequences function of the Software Excel. The total duration of the intraoral phase was 14 days (Fig. 1) with a 1-week wash-in period preceding it, as previously described^20–22^.

The volunteers were additionally informed to avoid consuming high fluoride-containing food, such as fluoridated salt, green/black tea and fish. At the beginning of each phase, the volunteers were also extensively trained to follow the study instructions. The intraoral appliances were worn during the whole day except for meals and oral hygiene. Through the whole study and additionally in the wash-in phase, individual oral hygiene was performed with a fluoride toothpaste (NaF, 1.450 ppm F^−^, Colgate Total 12, São Paulo, Brazil), without the appliances in situ. After meals, 20 min were elapsed before mouth appliance reinsertion.

### Sample size

The sample size was calculated based on the results of a previous study evaluating the inhibition of caries progression after resin infiltration in situ^23^. According to that, mean enamel mineral loss after the intraoral phase of 6261 (SD = 2009) vol% x µm for the negative control and 2559 (SD = 1178) vol%x µm for the infiltrated group would be expected. The tooth was considered as experimental unit and to obtain a power of 80%, a sample size of fourteen enamel blocks per treatment group was calculated (two-sided, α = 0.05). In order to account for a possible dropout, 15 specimens/groups were then included in the experiments.

Fifteen young adult volunteers (9 women, 6 men, aged 18–22 years), were recruited for the study. All subjects lived in Fortaleza, Brazil (fluoride concentration in tap water of approximately 0.8 mg/l) and met the following inclusion criteria: the ability to wear an intraoral palatal device for 24 h per day, except at eating and brushing times; no evidence of active caries, or periodontal disease; no signs of decreased salivary flow rate and the ability to comply with the study protocol. Exclusion criteria were any medical condition potentially interfering with the subject’s health; use of orthodontic appliance; pregnancy or breastfeeding and absence of compliance to the study protocol.

### Specimens

Seventy-five enamel blocks of 4 × 4 × 2 mm, disinfected as previously described^24,25^ were cut (Isomet, Buehler, Illinois, USA) from labial central area of bovine incisor crowns (teeth donated from a slaughterhouse, Fortaleza, Brazil). The enamel surface was serially polished using silicon carbide papers (grits #800, #1200, and #4000, Buehler, USA) under water cooling and polishing cloths with a 1-µm diamond suspension (Buehler, USA). After each polishing steps, the specimens were washed in ultrasonication bath containing deionized water for 3 min. The surface microhardness of all enamel blocks was determined with a Knoop indenter (6 indentations in the center of the specimens: 25 g load for 15 s), as previously described by Zero et al.^26^. Seventy-five enamel specimens were selected (average knoop hardness number (KHN): 252.69 ± 50.53 KHN), after discarding 6 blocks that presented outlier values. All specimens were examined under a stereo microscope to ensure the absence of hypomineralized areas or surface defects^27^ and were stored at 100% of humidity in a box containing deionized water, throughout the study, except for the time they were in situ.

### Artificial caries lesions

All selected specimens were all were laterally protected with acid resistant varnish (Colorama-CEIL, São Paulo, SP, Brazil), leaving only the labial enamel surface of the specimens exposed. Afterwards they were submitted to a well-stablished pH-cycling model for 8 days, in order to create artificial white-spot lesions, as previously described^28^. Shortly, each cycle consisted of 4 h immersion in the demineralization solution followed by 20 h in the remineralization solution at 37 °C. The demineralization solution was composed of (50 mmol/lactate buffer pH 5.0, containing 1.28 mmol/L Ca, 0.74 mmol/L Pi and 0.03 μg F/ml prepared to form the Ca(NO_3_) salt 2.4 H_2_O, KH_2_PO_4_ and NaF) (100 ml/16 mm^2^ of enamel. And the chemical composition of remineralization solution was 1.5 mmol/Ca, 0.9 mmol/P, 150 mmol/KCl and 0.05 μgF/mL in 0.01 mmol/L Tris buffer (pH 7.0 / 37° C) (2.5 ml solution/mm^2^ enamel surface)^28^. Twice a day (before and after immersion in the demineralization solution), the blocks were washed with deionized water for 5 min and under agitation, and on the fourth day, the demin- and remineralization solutions were renewed with fresh ones. After the end of the pH-cycling, the blocks were kept immersed in remineralization solution for 24 h^28^.

### Resin infiltration of caries lesions

After lesion formation all specimens were washed in running water and dried with sterile gauze. Subsequently micro-invasively treatment by the application of resinous infiltrating agent (ICON, Dental Milestones Guaranteed DMG, Hamburg, Germany) was carried out, according to the manufacturer’s protocol and as previously described^29^.

### Surface polishing protocols (treatments)

After that, the specimens were randomly divided, using an Excel-Sheet with random sequence of numbers, into five groups receiving the following polishing treatments:

- No polishing, as a negative control group (NC)
- polishing with aluminium oxide flexible disks (Al_2_O_3_-Disks), Sof-Lex Pop On
- polishing with silicon carbide tips (SIC-Tips), Astropol
- polishing with silicon carbide brush (SIC-Brush), Optishine
- polishing silicon carbide polyester strips (SIC-Strips), Epitex

All polishing protocols were performed by only one operator, for the same amount of time, in a pre-tested manner as well as following the manufacturers’ instructions. Further details are described in Table 1.

**Table 1:** Detailed description of polishing groups, trademarks and adopted application protocols. Grit sizes are provided for all instruments in µm.

Groups	Polishing systems	Protocols*
NC	Negative control	Only washing with water for 30 s
	No polishing	
Al_2_O_3_-disks	Aluminium oxide flexible disks	Discs: light orange (thin), grit size: 5.72 μm
		Yellow (ultrafine), grit size: 1.68 μm
	Sof-Lex Pop On (3 M–ESPE, Minnesota, USA)	Movements: intermittent, unidirectional
		Time: 15 s each, in total 30 s
		Application of air and water jets between one disk and another
SIC-tips	Silicone carbide tips	Tip: pink silicone tip, Silicon carbide, aluminium oxide, titanium oxide, and iron oxide, grit size: 3.5 μm
	Astropol (Ivoclar-Vivadent, Schaan, Liechtenstein)	Movements: intermittent, unidirectional with abundant water
		Time: 30 s
SIC-brush	Silicon carbide brush	Brush: silicon carbide abrasive particles embedded in the bristles, single-step system, grit size: 0.4 µm
	Optishine (KERR, California, EUA)	Movements: Intermittent, unidirectional with abundant water
		Time: 30 s
SIC-strips	Silicon carbide polyester strips	Sandpaper: fine (Gray), grit size: F-800 ca. 6.5 µm Extra Fine (Pink), grit size: F-1200 ca. 3.0 µm
	Epitex—GC Corporation, Bunkyo-ku, Tokyo, JAPAN	Movements: Back and forth
		Time: 15 s each, in total 30 s

*For all protocols, the discs, tips, brushes, or strips were changed after the polishing of each 3rd specimen.

### Surface roughness

**Figure 2:**
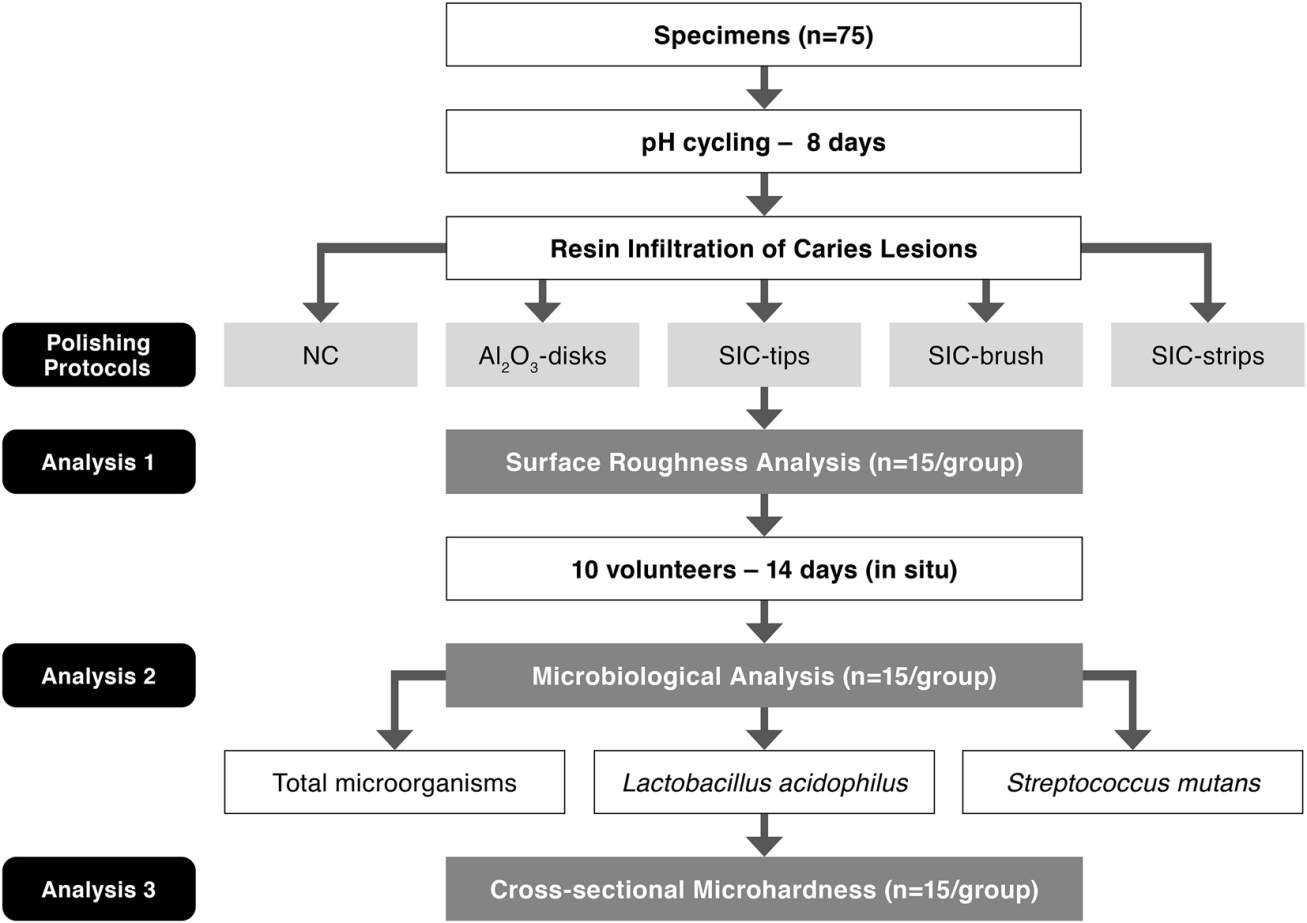
Flow-chart of the in situ study. Analysis of surface roughness was performed before the in situ phase and microbiological as well as cross-sectional microhardness afterwards. Samples distributions for each analysis (n = 15/group) by each analysis.

In order to evaluate the effect of the different polishing systems on the surface characteristics of infiltrated enamel all specimens were subjected to a surface roughness analysis (Fig. 2) using a contact profilometer (Hommel-Etamic W10, Schwenningem, Germany). The device has an accuracy of 0.01 µm, and the diamond stylus has a radius of 5 µm. Measurements were done in the center of the specimens at a constant speed of 0.15 mm/s and under a force of 0.8 mN. The average roughness values (Ra) were obtained after three successive scannings, located at 100 μm distance to each other, as previously described^30,31^. An arithmetic mean of the values measured was considered the value (Ra) in μm for each specimen.

### Palatal appliances

After hard and soft tissue examination intraoral palatal devices of acrylic resin were prepared for each volunteer. In each palatal device, 5 niches were created, where the specimens containing the infiltrated caries lesions were fixed (Fig. 2). A free space of 1.0 mm over the specimens and the plastic mesh covering then was preserved, in order to allow for biofilm accumulation. The mesh net also protected the biofilm against mechanical disturbances^21,24^.

### Intraoral phase

To trigger a cariogenic challenge, the volunteers dripped 10% sucrose solution over each specimen 8 times a day, for 14 days (Fig. 2). In total the volunteers wore the intraoral appliances for 5 phases of 14 days each, with continuous in situ exposure except during meals and oral hygiene, meaning a total wearing time of 22–23 h/day.

### Cross-sectional microhardness analysis

After the in situ cariogenic challenge and biofilm collection, a longitudinal section was performed in the center of each specimen. One of the halves of each specimen was embedded in acrylic resin, the cut surface being exposed. The cut surfaces were subsequently serially polished as described in the specimen preparation section and the cross-sectional microhardness was measured. For this, two lines of fourteen Knoop indentations were done at 25 g load for 5 s^20,32,33^. The two lines of indentations were located 100 μm apart from each other^34^. Within each sequence, the first ten indentations were spaced 10 µm from the previous one and the last ones were spaced at 20 μm distance (10, 20, 30, 40, 50, 60, 70, 80, 90, 100, 120, 140, 160, 180 μm distance from the enamel surface)^35^. The mean Knoop hardness number (Kg/mm^2^) of the two rows at each distance from the surface were then averaged and plotted over the lesion depth (µm). The data set of each artificial caries lesion was curve-fitted^33^, and the area under the lesion tracing was calculated and subtracted from the area under the curve of sound enamel mean values to give the parameter (ΔS, Kg/mm^2^ x µm)^32^. As a linear relationship between cross-sectional microhardness and mineral content of enamel measured by transversal microradiography methods has been demonstrated^33^.

### Microbiological analysis

Microbiological analysis of the biofilm formed on each specimen was performed, in order to evaluate the effect of polishing of the resin infiltrants on the adhesion of caries-associated bacteria species^36^. A biofilm sample from each specimen was collected. The obtained suspension (addition of 0.9% NaCl solution (1 ml/mg of biofilm) was diluted in decimal series and inoculated in triplicate in blood agar culture media to determine total microorganisms (TM), and MSKB (90 g *mitis salivarius* dehydrated agar; 1 ml 1% potassium tellurite; 20% w/v sorbitol; 1ug/ml kanamycin monosulfate; 0.1 U/ml bacitracin) for counting *Streptococcus mutans* and Man Rogosa Sharpe agar for counting Lactobacillus *acidophilus* (kept for 48 h in an oven at 37 °C with a partial pressure of 5% CO_2_) for 48 h, the ratio of colony forming units (CFU) per milligram of biofilm was established (CFU/mg).

#### Scanning Electron Microscope (SEM) analysis

After the in situ phase, representative blocks of the treatment groups were subjected to SEM analysis. The specimens were cleaned in a ultrasonic bath, rinsed with ethanol (96%), dried and subsequently mounted on metallic stubs. After gold sputtering-coat they were examined under a SEM (Quanta 450 FEG, FEI Company^®^, Oregon, EUA) at 1000 ×, 5000 × and 10,000 × magnification. The images were obtained using an Everhardt-Thornley detector, the accelerating voltage was 20 kV and the working distance 13, 2 mm.

### Statistical analysis

Normal distribution of all collected data was assessed using the Shapiro-Wilks statistical test as 5% significance level. Since the average surface roughness data were not normally distributed, they were analysed using a Kruskal–Wallis test and post hoc comparisons. Data obtained from the cross-sectional microhardness and ΔS analyses were analysed by means of a one-way ANOVA and Tukey’s post hoc comparisons to detect differences between the treatment groups. For the microbiological analysis, the variables of colony forming units (CFU) of each group were logarithmically transformed and then analysed by means of ANOVA and Tukey-test for post hoc comparisons. All tests were conducted at a significance level of 5% using the Statistical Package for the Social Sciences (SPSS 22.0) for Windows.

## Results

### Surface roughness

**Figure 3:**
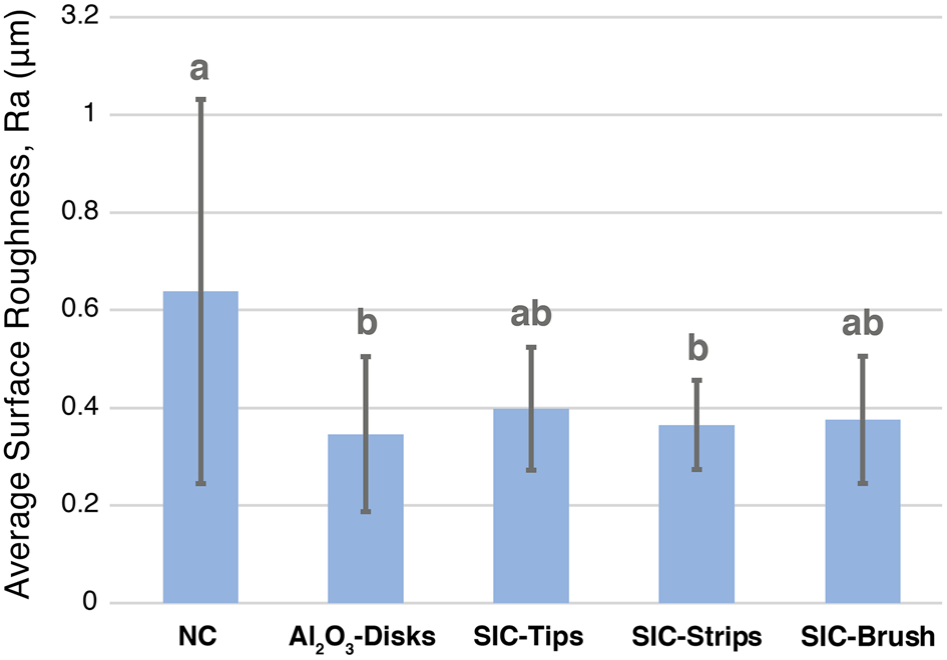
Means and standard deviations of average surface roughness (Ra) of enamel after use of the different polishing protocols.

The results of the surface roughness analysis after each polishing protocol are shown in Fig. 3. In general, there was a significant effect of the type of polishing protocol on enamel surface roughness (p = 0.01). Statistically significant difference was found between NC and Al_2_O_3_-disks (p < 0.01) and between NC and SIC-strips (p = 0.04). Among the test groups, meaning the different polishing protocols, no significant difference was observed (p > 0.05).

### Cross-sectional microhardness analysis

**Figure 4:**
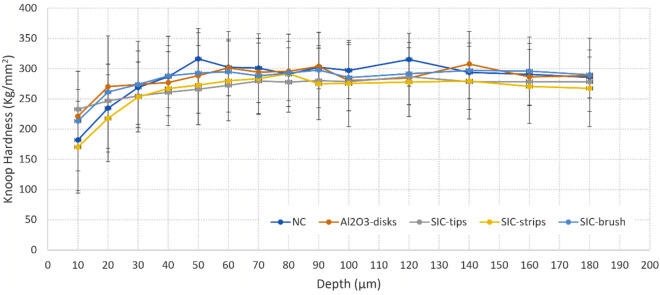
Mean Hardness (kg/mm^2^) profile of enamel after the intraoral period according to distance (μm) from the surface for the different groups (bars denote SD).

Regarding protection from demineralization under the in situ cariogenic challenge, the lowest ΔS mean (Kg/mm^2^Xµm) was observed for Al_2_O_3_-Disk (5931.9/CI 1769.3–10,094.6), however none of the groups were significantly different to NC (6983.3/CI 4246.1–9720.5, p > 0.05) (Table 2, Fig. 4).

**Table 2:** Means and 95% confidence intervals of the cross-sectional microhardness (Knoop hardness) measurements.

	Groups	Delta S		*
		Mean (Δs, Kg/mm^2^ x µm)	95% CI	
Negative control	NC	6983.3	(4246.1–9720.5)	a
Aluminium oxide flexible disks	Al_2_O_3_-disks	5931.9	(1769.3–10,094.6)	a
Silicon carbide tips	SIC-tips	7460.5	(4053.1–10,867.9)	a
Silicon carbide brush	SIC-brush	7783.9	(3466.3–12,101.4)	a
Silicon carbide polyester strips	SIC-strips	8845.5	(4733.5–12,957.5)	a

*ANOVA statistical analysis showed no significantly difference between the groups (p = 0.783).

### Microbiological analysis

**Figure 5:**
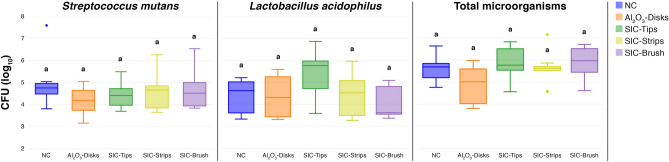
Box-plot diagrams of the colony forming units of total microorganisms, *Streptococcus mutans* and *Lactobacillus acidophilus* recovered from the plaque formed over the specimens.

The logarithms of the microbiological counts after polishing in the in situ microbiological assay can be found in Fig. 5. In all the bacterial counts investigated, namely *Streptococcus mutans*, *Lactobacillus acidophilus* and total microorganisms there was no statistically significant difference between the groups (p = 0.89, p = 0.37, p = 0.58 respectively).

#### Scanning Electron Microscope (SEM) analysis

**Figure 6:**
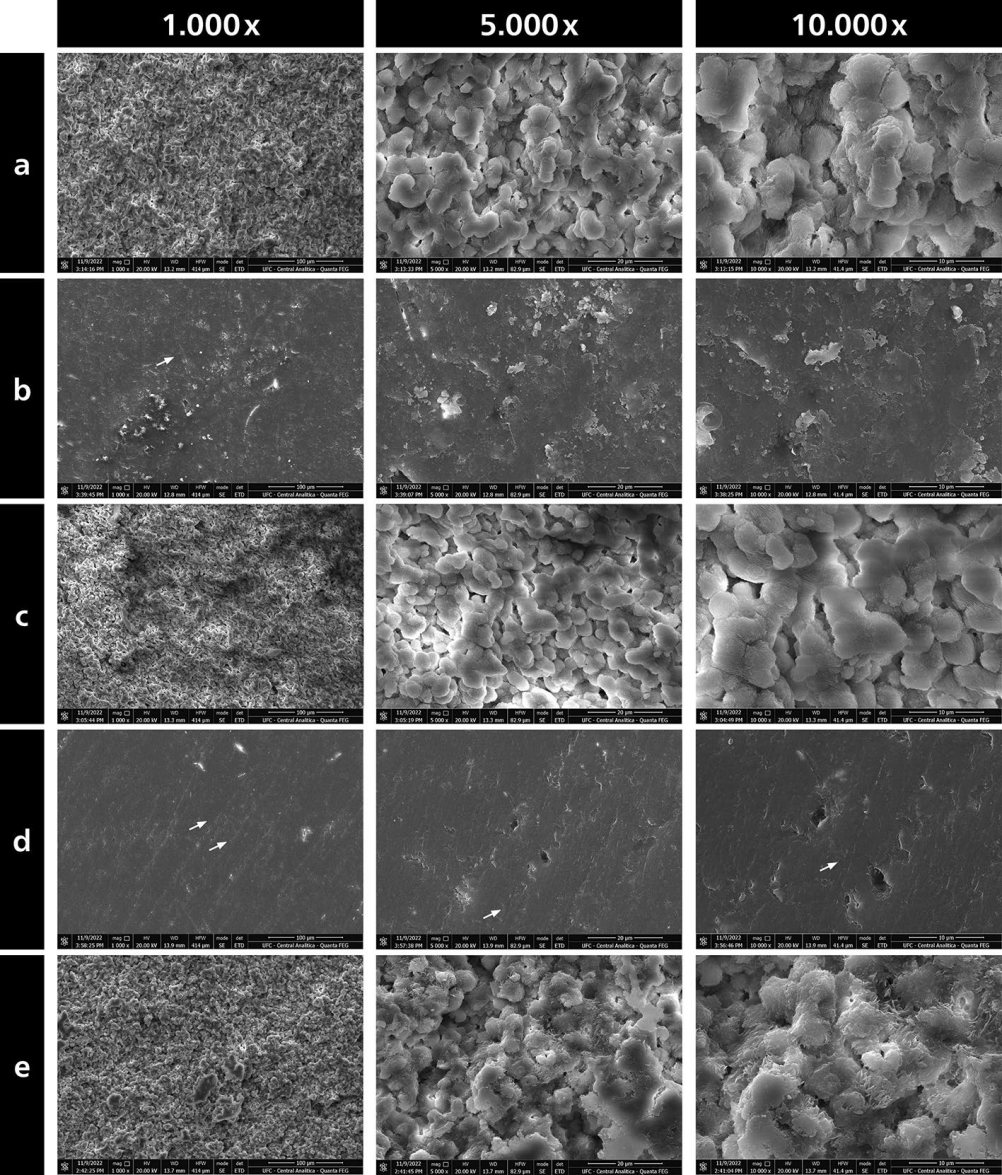
Representative scanning electron microscopy images of the enamel surfaces after the different polishing protocols and after 14 days in situ (Magnifications: 1.000 X, 5.000 X, 10.000 X). White arrows indicate groves, valleys and scratches on the enamel surfaces caused by the polishing procedures, which were still present after the intraoral caries challenge. Moreover, the group polished with Al_2_O_3_-disks showed the least amount of biofilm still present over enamel surface, which relates well to the previous results in which this group also showed the least amount of total bacterial counts, the lowest surface roughness, and the lowest demineralization. Although all samples were previously cleaned in an ultrasonic bath a mixture of polishing debris and bacterial biofilm was still shown for the negative control as well as for SIC-tips and SIC-strips polishing approaches. (**a**) SIC-tips, (**b**) SIC-brush, (**c**) SIC-strips, (**d**) Al_2_O_3_-disks, (**e**) NC.

The SEM analysis of the enamel surfaces after the different polishing protocols and after 14 days in situ showed a wide range of characteristic modifications (Fig. 6). In general, most of the groups still showed some debris of the polishing procedures, while only groups SIC-brush and Al_2_O_3_-disks presented groves, valleys and scratches caused by the polishing procedures and which were still present even after the intraoral caries challenge. Moreover, the group polished with Al_2_O_3_-disks showed the least amount of biofilm still present over enamel surface, which relates well to the previous results in which this group also showed the least amount of total bacterial counts, the lowest surface roughness, and the lowest demineralization. It is interesting to note that, although all samples were previously cleaned in an ultrasonic bath, a mixture of polishing debris and bacterial biofilm was still shown for the negative control as well as for SIC-tips and SIC-strips polishing approaches.

## Discussion

The present study evaluated for the first time the efficacy of polishing protocols after resin infiltration using an in situ model. The results showed that infiltrated and subsequently polished enamel lesions do not demineralize significantly less compared to unpolished lesions. Moreover, bacterial adhesion of caries-associated bacteria, like *Streptococcus mutans* and *Lactobacillus acidophilus,* but also of total bacterial was not significantly reduced, partially rejecting the formulated study hypothesis. However, average surface roughness of resin-infiltrated enamel caries lesions was significantly reduced after polishing with Al_2_O_3_-Disks and SIC-Strips confirming what was initially hypothesized; at least for these two protocols.

Currently, there are little evidence from in vitro and only limited indirect evidence from clinical studies as regards the effect of solely resin infiltration or infiltration followed by several polishing protocols on surface roughness and bacterial adhesion of infiltrated carious lesions. The significantly reduced surface roughness observed after polishing with Al_2_O_3_-Disks and SIC-Strips are not totally in agreement with the only one in vitro study investigating polishing of resin-infiltrated lesions. In the study from Mueller, et al.^15^ polishing the incipient lesions with only one type of polishing system, namely polishing strips (coarse, medium, fine and superfine, each: 15 s, total time:60 s), after light curing of the resin infiltrant resulted in average surface roughness means not significantly different to the non-polished groups. The differences in the results may be related to the fact that the caries lesions in the mentioned study^15^ were much deeper than in our study, implicating in the presence of much larger surface pores, which may have resulted in much higher initial surface roughness and a more difficult baseline situation for achieving a high degree of polishing. Furthermore, the study design may have been also an influence factor, as the present study the cariogenic challenge was performed in situ, including the influence of the salivary pellicle, and in the previous study only in vitro simulations were performed.

As regards the bacterial adhesion to the best of our knowledge this was the first study investigating bacterial adhesion of two caries-associated bacteria (*S. mutans* and *L. acidophilus*) in situ and after polishing of resin-infiltrated lesions. In fact, the only previous study investigating bacterial adhesion after a resin infiltration procedure, did not included any polishing afterwards, while investigating only one bacteria species, namely *Streptococcus mutans*^18^. In this way the approaches are not directly comparable, but anyway also without polishing there was a slightly reduced adhesion of *S. mutans*, which was not significantly different to the non-treated control^18^. This indicates a tendency towards absence of an impact of resin infiltration on biofilm formation. Nevertheless, a better understanding of the influence of resin infiltration on bacterial adhesion is still needed and must be further investigated. Moreover, the present results must be interpreted with caution as other cariogenic species play a key role in a cariogenic biofilm or in the transition to dysbiosis of the microbiome^1^.

Another aspect interest to observe is that even in the groups causing a significant decrease in surface roughness, like Al_2_O_3_-Disks and SIC-Strips, this decrease was not accompanied by a decrease in the bacterial adhesion over these more polished surfaces. This was somehow unexpected, but at the same time shows that surface roughness is not the solely topography parameter impacting bacterial adhesion. Indeed, also surface free energy, charge, polarity and morphology has been shown to substantially influence bacterial adhesion and growth^6,7^. Moreover, the means of average surface roughness (Ra) in the present study varied from 0.3 to 0.6 µm, slightly above a limit of 0.2 µm, over which surfaces were supposed to favour higher bacterial adhesion^10^. This is also in agreement with a recent systematic review contesting this threshold value for bacterial adhesion over dental material surfaces^11^. In addition factors like type of material and study design may also influence bacterial adhesion, with studies including formation of salivary pellicle*, *in situ or in vivo, having a clear advantage of better simulating the clinical conditions, where early colonizers first attach to salivary pellicle and the latter may also modify the surface of properties of the materials^6,12^.

It can be speculated that one of the reasons for the absence of influence of surface roughness on bacterial adhesion in the present study may be related to the presence of a naturally formed salivary pellicle over the enamel surfaces. Since there is evidence that the presence of the pellicle may level out grooves and valleys of a rough enamel surface^6,7^. However, bacterial adhesion to dental and material surfaces is a complex multi-stage process, which is up to now not completely understood. There is only a clear understanding that surface topography may influence the near-surface micro-environment and therefore plays a important role in bacterial adhesion^7,11^. Therefore, future investigations could systematically evaluate also other topographic parameters for improving the understanding of the biofilm-surface interactions.

For sure the use of bovine enamel samples provides similar but not equal demineralization responses as human enamel and thus represent a limitation of the results shown. However, at the same time, the study design allowed for the first time an analysis of the influence of polishing of resin-infiltrated incipient caries lesions on surface roughness, bacterial adhesion, and the caries protective effect within the oral cavity and under cariogenic conditions. This represents an important advantage of the model as, among other reasons, it is well known that bacterial growth over enamel or restoration surfaces in highly influenced by the presence or absence of a naturally formed salivary pellicle^6^. Thus, a better clinical simulation of the biofilm film formed clinically was achieved. Nonetheless, the clinical efficacy of polishing resin-infiltrated caries lesions must still be investigated under a more severe cariogenic model, including longer sucrose challenges and/or longer periods in situ*.*

Furthermore, the comparative effectiveness of the resin infiltrate against newly introduced treatments for arresting or remineralizing carious white-spot lesions, like the biomimetic approaches using hydroxyapatite^37,38^ and self-assembling peptide P11-4^39^ therapies should be further investigated in the future. Both in relation to their remineralizing and their effects on enamel surface properties as well as bacterial colonization.

As regards the methods used, it is important to notice that cross-sectional microhardness is also a wide-used and valid method of measuring changes in enamel mineral content (i.e. demineralization). The reason for that is because a linear correlation between values of cross-sectional microhardness and volume mineral loss (%vol x µm) has been demonstrated both in vitro^33^ as in situ^40^. Transversal microradiography is the current gold standard technique, and this clear correlation indicates that cross-sectional microhardness is a sensitive and reliable method for assessing mineral loss of incipient carious lesions. As regards the microbial counts through cultivation methods and counting of colony forming units, it is reasonable to say that although currently more modern molecular biological methods are available, for several research questions the classic cultivation methods still provide very useful information^24,36^. As both microorganisms tested here grow well in the cultivation plates and the microbiological analyses were just an ancillary method in the current work, it surely fulfilled the nees. For future studies it might be interesting though to include also other analyses like fluorescence microscopy or investigations of the 16S rRNA gene.

Although the present results showed that polishing neither influenced the caries-protective effect of resin infiltration nor the bacterial adhesion, from a clinical point of view and specially for labial white-spot lesions infiltrated for masking the whitish appearance, it is still probably more reasonable to polish then anyway. Data from previous clinical studies on this application showed that labial lesions may suffer from long-term staining, when not polished^41–43^. Moreover, contrary to the clinical studies on approximal lesions, which all did not report polishing procedures^29,44–47^, for labial lesions most of the clinical studies have been performed including polishing steps after light curing of the infiltrant^41^. In the study from Kobbe et al. 2019 for example polishing with disks (Sof-Lex) and a polishing brush (Okklubrush) were included and resulted in non-staining in the short-term follow-up.

It was shown for the first time in a pre-clinical intraoral model that some polishing protocols influenced surface roughness, while not significantly affecting neither bacterial adhesion nor enamel demineralization, under the conditions of this study. In this way, not contradicting the clinical recommendation of only removing surplus infiltrant material before light curing. However, one must bear in mind that this question of plaque stagnation over resin-infiltrated should be further investigated either in vivo or with in situ models including longer and more aggressive cariogenic challenges as well as including other polishing protocols before a clinical recommendation as regards the polishing need after infiltration of both buccal as well as approximal caries lesions can be made.

Thus, in conlcusion polishing protocols including Al_2_O_3_-Disks and SIC-strips significantly decreased average roughness of resin-infiltrated caries lesions in enamel. However, none of the polishing protocols could significantly decrease the bacterial counts nor resulted in significantly less demineralization than the non-polished control. From a clinical point of view and specially for labial white-spot lesions infiltrated for masking the whitish appearance, it is still probably more reasonable to polish then anyway.

## Data Availability

The data collected and analysed in this paper is available from the corresponding author upon reasonable request.
